# Recovery of Metal Ions (Cd^2+^, Co^2+^, and Ni^2+^) from Nitrate and Sulfate on Laser-Induced Graphene Film Using Applied Voltage and Its Application

**DOI:** 10.3390/ma17122965

**Published:** 2024-06-17

**Authors:** Xiu-man Wang, Tong Su, Yujun Chai

**Affiliations:** 1 State Key Laboratory of Mountain Bridge and Tunnel Engineering, Chongqing Jiaotong University, Chongqing 400074, China; 2 School of Civil Engineering, Chongqing Jiaotong University, Chongqing 400074, China; 3 College of Chemistry and Materials Science, Hebei Normal University, Shijiazhuang 050024, China; 20190801199@cqu.edu.cn (T.S.); yjchaiwn@gmail.com (Y.C.)

**Keywords:** recovery, transition metal, LIG/PI film electrode

## Abstract

The urgent removal of Cd, Co, and Ni from nitrate and sulfate is essential to mitigate the potential risk of chemical pollution from large volumes of industrial wastewater. In this study, these metal ions were rapidly recovered through applying voltage on nitrate and sulfate, utilizing laser-induced graphene/polyimide (LIG/PI) film as the electrode. Following the application of external voltage, both the pH value and conductivity of the solution undergo changes. Compared to Co^2+^ and Ni^2+^, Cd^2+^ exhibits a lower standard electrode potential and stronger reducibility. Consequently, in both nitrate and sulfate solutions, the reaction sequence follows the order of Cd^2+^ > Co^2+^ > Ni^2+^, with the corresponding electrode adsorption quantities in the order of Cd^2+^ > Co^2+^ ~ Ni^2+^. Additionally, using the recovered Co(OH)_2_ as the raw material, a LiCoO_2_ composite was prepared. The assembled battery with this composite exhibited a specific capacity of 122.8 mAh g^−1^, meeting practical application requirements. This research has significance for fostering green development.

## 1. Introduction

Large amounts of residual metal ions remain in industrial wastewater. Once metal ions contaminate water or soil, they pollute the environment and affect people’s health [1]. Therefore, an efficient and environmentally friendly disposal method for treating polluted waterbodies is an urgent issue.

Researchers have employed various methods to recover valuable metal ions from waste batteries, including leaching, chemical reduction, metallurgy, and other techniques [2,3,4,5]. Meshram et al. [6] reviewed the recovery of metals using various acids, which unfortunately led to secondary pollution caused by the chemical agents. In recent decades, electrosorption or capacitive deionization (CDI), which rapidly pushes the migration of ionics using electrical force, has been widely utilized for the recovery of metal ions in wastewater due to its low cost, ease of control, and absence of secondary pollution from the chemical agents [7,8,9]. The adsorption electrode serves as the central component of the electric deionization device, playing a pivotal role in determining the device’s adsorption capacity [8,10]. Electrode materials featuring a carbon network structure have been extensively researched due to their high specific surface area, well-balanced pore size distribution, exceptional electrical conductivity, and favorable adsorption properties [9,11,12,13]. Li et al. [14] reported on the removal of Ni^2+^ ions using an α-MnO_2_/carbon fiber paper composite, which exhibited pseudocapacitor properties, facilitating electron/charge transfer. Xue et al. developed a nitric acid-modified activated carbon electrode for the removal of Co^2+^, Mn^2+^, and Ni^2+^ ions [15]. Coconut-shell activated carbon, activated by CO_2_, served as the electrode, demonstrating a Ni removal capacity of 5.32 mg g^−1^ [16]. This implies that modifying carbon materials with various functional groups can effectively be used to adsorb metal ions.

Laser-induced graphene (LIG), as an easily obtainable carbon material, has been utilized across various fields due to its high thermal stability, excellent conductivity, rich 3D porous network, and abundant functional groups [17]. In our previous research, employing LIG as an electrode material exhibited outstanding capability in recovering rare earth ions [18]. Li et al. reported that a Co_4_S_3_^−^ modified LIG electrode exhibited a high adsorption capacity of 2702.79 mg g^−1^ for UO_2_^2+^ [19], indicating its potential as an absorbent electrode.

To our knowledge, the use of LIG films as electrodes for recovering Cd^2+^, Co^2+^, and Ni^2+^ ions has not been reported previously. Therefore, the recovery of Cd^2+^, Co^2+^, and Ni^2+^ ions in nitrate and sulfate forms was investigated in this work. The standard potential of Cd (E^θ^ = −0.403 V vs. SHE) is lower than that of Co (E^θ^ = −0.277 V vs. SHE) and Ni (E^θ^ = −0.257 V vs. SHE), indicating the easy recovery of cadmium ions under identical solution concentrations. Due to the superior oxidation capabilities of nitrate ions, the ions recovered from nitrate solutions are mainly hydroxides, whereas the products recovered from sulphate solutions are metals. Furthermore, the cobalt hydroxide recovered could serve as a raw material for the preparation of lithium-ion electrode materials. This has significance for fostering green development.

## 2. Materials and Methods

### 2.1. Materials and Characteristics

Co(NO_3_)_2_, Ni(NO_3_)_2_, Cd(NO_3_)_2_, CoSO_4_, NiSO_4_, and CdSO_4_ were procured from Sinopharm Group Chemical Reagent Co., Ltd, Shanghai, China. A 100 μm polyimide (PI) film was supplied by Texiang Electrical Insulation Material Company, PRC, Tianjin. Ethanol was purchased from Chongqing Colon Chemical Co., Ltd., Chongqing, China. Deionized water specifically prepared for laboratory use was employed. The aforementioned chemicals are of analytical purity and were used without any further treatment.

The structure and morphology of the product were characterized using X-ray diffractometry (XRD, SmartLab, Shanghai, China) and field-emission scanning electron microscopy (FESEM, Hitachi S-4800, Tokyo, Japan). The bonds in the sample were analyzed using Fourier-transform infrared spectroscopy (FTIR, Thermo Scientific, Suzhou, China) and X-ray photoelectron spectroscopy (XPS, Thermo Fisher Scientific Escalab QXi, Waltham, America). The pH of the solution was determined using a PHS-3C pH acidity meter. The concentration of ions in solution during the application of external voltage was measured using atomic absorption spectrometry (AAS, TAS-990 SUPER, Weizhou, China). The electrochemical performance of the electrode was carried out using VMP3 multi-channel electrochemical impedance system.

### 2.2. Preparation of LIG/PI Film Electrodes via Laser Scribing

**Figure 1:**
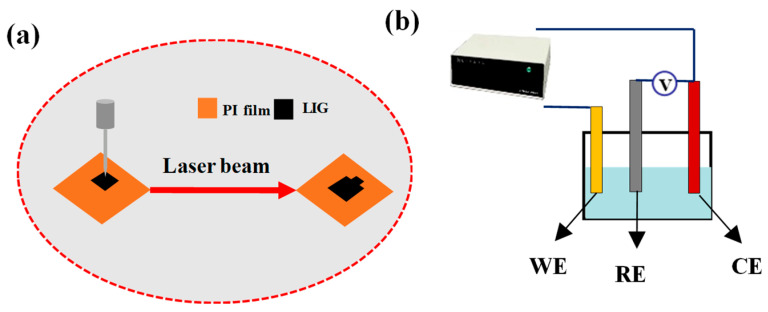
(**a**) Schematic diagram of the fabrication of LIG/PI film electrodes. (**b**) Schematic diagram of the three-electrode system.

Firstly, commercial PI films were cleaned with ethanol and deionized water. Subsequently, a 100 μm thick PI film was affixed to a glass plate, and a CO_2_ laser with a wavelength of 10.6 μm was utilized to carve the PI film, resulting in the production of an LIG/PI film electrode. The scanning speed and scanning power of the laser engraving machine were 100 mm/s and 6.6 W, respectively. Finally, an LIG/PI thin film electrode with a size of 2.5 × 1 cm^2^ was obtained. Figure 1a shows the preparation process of the LIG/PI thin film electrode.

In this experiment, a traditional three-electrode system (Figure 1b) was employed to the simulated solutions containing 0.005–0.02 M Co^2+^, Ni^2+^, and Cd^2+^. The LIG/PI film served as the working electrode, while a Pt electrode was utilized as the counter electrode. An Ag-AgCl electrode was employed as the reference electrode. Upon applying a voltage to the reference electrode, the ions were recovered. The formulas for calculating the adsorption capacity (Q) and removal rate (W) are provided in detail in the Supplementary Materials.

### 2.3. Application of Recovered Co(OH)_2_

LiCoO_2_ was prepared using the sol-gel method. First, the recovered Co(OH)_2_ and citric acid in a stoichiometric ratio of 1:2 were dissolved in 50 mL distilled water; then, the mixture was heated in an oil bath for 1 h at 80 °C with stirring. A certain amount of LiOH was added; meanwhile, an ammonia solution was used to adjust the pH of solution. The mixture was dried at 120 °C to evaporate the water. Finally, it was annealed at 800 °C for 8 h to obtain LiCoO_2_.

A slurry was prepared by mixing LiCoO_2_ (80 wt%), Super P (10 wt%), and polyvinylidene fluoride (PVDF, 10 wt%) in N-methyl-pyrrolidone (NMP). Subsequently, the half-cell was assembled and measured following the procedures outlined in previous studies [1].

## 3. Results

### 3.1. Change in pH and Conductivity after Applying the External Potential

**Figure 2:**
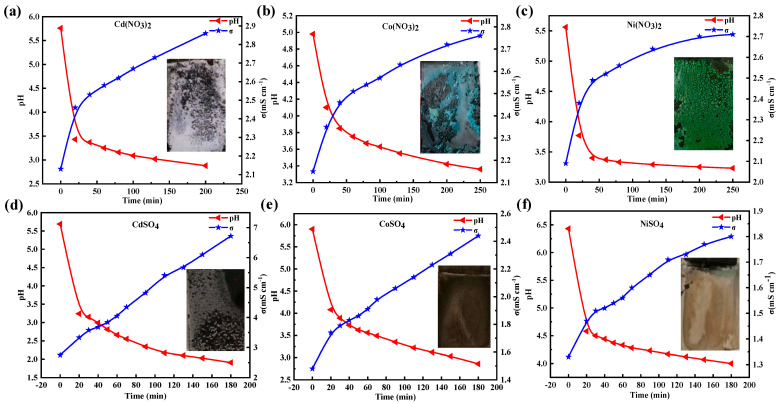
Changes in pH and conductivity after applying the potential (−1.2 V). (**a**) Cd(NO_3_)_2_, (**b**) Co(NO_3_)_2_, (**c**) Ni(NO_3_)_2_, (**d**) CdSO_4_, (**e**) CoSO_4_, (**f**) NiSO_4_.

Figure 2 illustrates the changes in pH and conductivity following the application of an external potential to a 0.02 M solution of Cd(NO_3_)_2_, Co(NO_3_)_2_, and Ni(NO_3_)_2_. When applying a potential of −1.2 V (vs. Ag/AgCl) to a 0.02 M solution of Cd(NO_3_)_2_, a silver-white product was obtained (inset of Figure 2a). In the Co(NO_3_)_2_ solution, both a gray product and a light blue product were obtained (inset of Figure 2b). And a green product was observed in the Ni(NO_3_)_2_ solution (inset of Figure 2c). Correspondingly, the pH decreased from ~6.0 to ~2.5, and the conductivity increased from ~2.0 to ~2.9 in these solutions. After the potential became −1.5 V, different color products were obtained, as shown in the inset of Figure S1. This illustrated a difference in the composition of products. Herein, a more obvious change in the pH and conductivity was detected. A higher applied voltage implies a strong electric driving force, which effectively influences the mobility of Co^2+^, Ni^2+^, and Cd^2+^ ions.

When applying a potential of −1.2 V to the sulfate solutions (Figure 2d–f), the variation in the pH and conductivity was notably significant in the CdSO_4_ solution (ΔpH = 3.78, Δσ = 3.97 mS cm^−^^1^). However, it was relatively small in CoSO_4_ (ΔpH = 3.04, Δσ = 0.96 mS cm^−^^1^) and NiSO_4_(ΔpH = 2.43, Δσ = 0.47 mS cm^−^^1^). Changing the potential to −1.5 V (Figure S1d–f) resulted in a dramatic change in pH (ΔpH = 4) and conductivity (Δσ = 8 mS cm^−^^1^) observed in the CdSO_4_ solution. It was smallest in the NiSO_4_ solution (ΔpH = 3, Δσ = 2 mS cm^−^^1^). These results illustrate that the reaction kinetics in sulfate and nitrate are different. The reaction ability in this solution follows the order of Cd^2+^ > Co^2+^ > Ni^2+^ under these conditions, disregarding the effect of anions.

### 3.2. Analysis of the Adsorption Product

**Figure 3:**
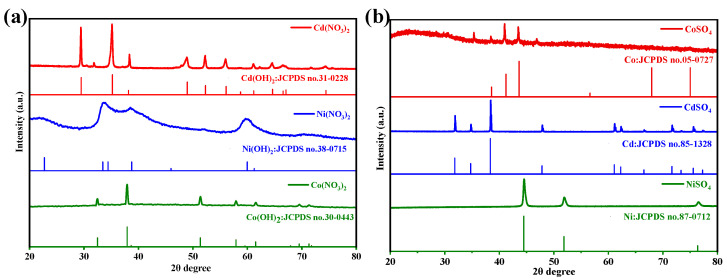
XRD pattern of electrode surface products in (**a**) nitric acid and (**b**) sulfate solutions with a voltage of −1.2 V.

**Figure 4:**
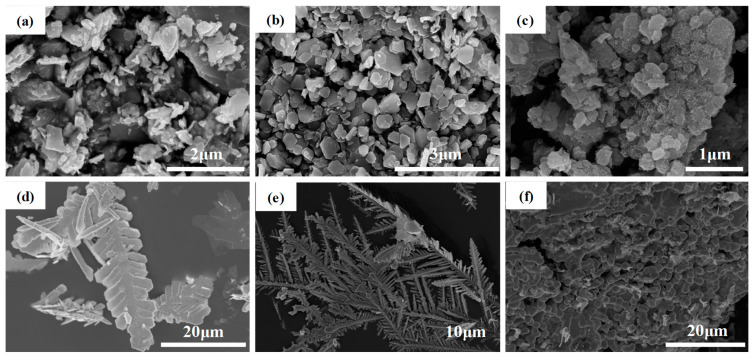
SEM images of the products in (**a**) Cd(NO_3_)_2_, (**b**) Co(NO_3_)_2_, (**c**) Ni(NO_3_)_2_, (**d**) CdSO_4_, (**e**) CoSO_4_ and (**f**) NiSO_4_.

The product compositions obtained on electrode surfaces after application of −1.2 V voltage in nitrate and sulfate solutions was determined using X-ray diffraction (XRD) analysis, as depicted in Figure 3. It can be observed that the sharp and strong peaks at 2*θ* = 18.7, 29.5, 35.1, and 59.7° belonged to Cd(OH)_2_ (JCPDS 31-0228) and the peaks at 2*θ* = 19.1, 32.5, 37.9, and 51.4° were identified as well-crystallized Co(OH)_2_ (JCPDS 30-0443) in nitrates [20,21]. However, the peaks around 2*θ* = 33.5, 38.5, and 59.7° were broad and poor, indicating the formation of amorphous Ni(OH)_2_/NiOOH (JCPDS 38-0715). The SEM further revealed that irregular blocks of Cd(OH)_2_ formed due to the rapid reaction (Figure 4a). A sheet-like structure of Co(OH)_2_ (Figure 4b) and an irregular rod-like shape of Ni(OH)_2_/NiOOH (Figure 4c) were generated, with the particles aggregating to form blocks. However, the adsorption products in the sulfate solutions consisted of metallic Cd (JCPDS 85-1328), Co (JCPDS 05-0727), and Ni (JCPDS 87-0712). Among them, metallic Cd and Co exhibited dendritic morphology, as depicted in Figure 4d,e. The morphology of Ni appeared as irregular spherical shapes (Figure 4f).

When the voltage was −1.5 V, hydroxides could be obtained in these nitrates (Figure S2). However, the morphology of the Cd(OH)_2_, Ni(OH)_2_/NiOOH and Co(OH)_2_ obviously changed (Figure S3). Although no change in the adsorbed products was detected from the XRD profiles in sulfate solution, the dendritic crystal became more obvious (Figure S4).

**Figure 5:**
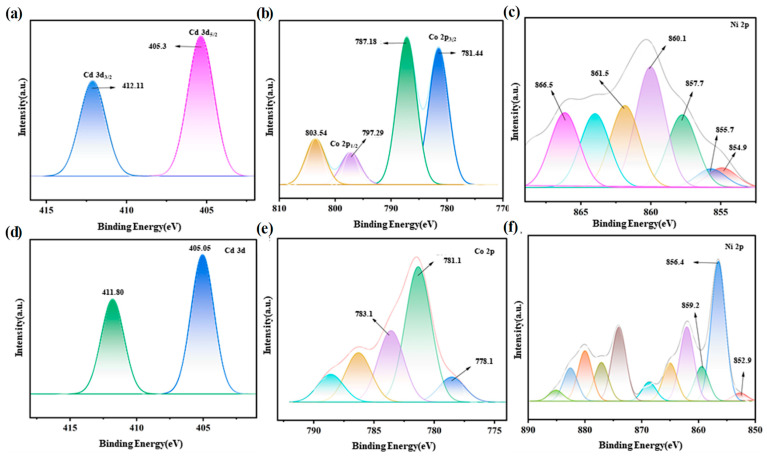
XPS the of product in (**a**) Cd(NO_3_)_2_, (**b**) Co(NO_3_)_2_, (**c**) Ni(NO_3_)_2_, (**d**) CdSO_4_, (**e**) CoSO_4_, and (**f**) NiSO_4_.

The chemical state of the product on the surface of the LIG film was analyzed using XPS, as shown in Figure 5. All XPS data were calibrated and fitted using PeakFit software 4.12. In the fitted spectra of products in Cd(NO_3_)_2_ solution (Figure 4a), two characteristic peaks were observed at 405.3 eV (Cd 3d_5/2_) and 412.11 eV (Cd 3d_3/2_), indicative of Cd^2+^ in Cd(OH)_2_ [22]. The position of the fitted peak in the Cd 3d spectrum of CdSO_4_ was identical to that of Cd(NO_3_)_2_, as zero-valent cadmium exhibits no characteristic peak. The results of XRD show that the adsorbed product of CdSO_4_ is Cd. This illustrates that besides the metallic Cd, trace of hydroxide existed. In the Co(NO_3_)_2_ solution, the peaks observed at 781.44 and 797.29 eV corresponded to Co 2p_3/2_ and Co 2p_1/2_ in Co(OH)_2_, respectively [23]. The satellite peaks at 787.18 and 803.54 eV, respectively, corresponded to the high spin state of Co^2+^ [24]. The fitting of the Co 2p spectra from CoSO_4_ (Figure 5e) exhibited significant differences compared to that from Co(NO_3_)_2_. Herein, characteristic peaks emerge at 778.1, 781.1, and 783.1 eV, respectively, corresponding to metallic Co [25]. The Ni 2p spectrum in Ni(NO_3_)_2_ was fitted with six characteristic peaks located at 854.8, 855.7, 857.7, 860.1, 861.5 and 866.5 eV, respectively. This indicates that the product is Ni(OH)_2_ or NiOOH, aligning with the reports in the literature [26]. The characteristic peaks of the Ni 2p spectra from NiSO_4_ (Figure 5f) were observed at 852.9, 856.4 and 859.2 eV, respectively, corresponding to metallic Ni.

### 3.3. Electrochemical Properties

**Figure 6:**
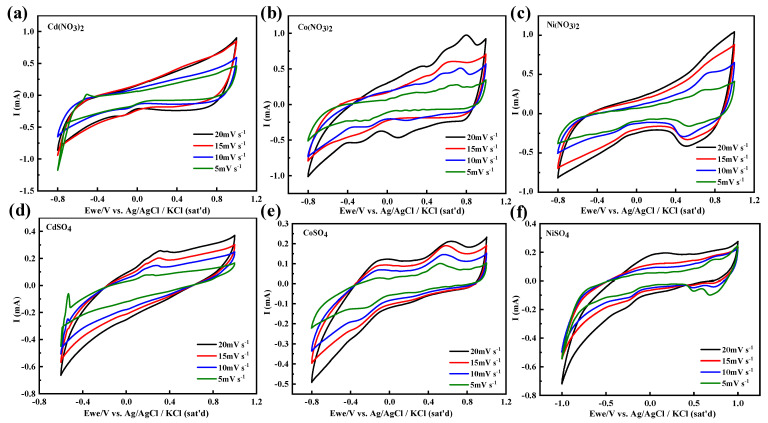
The CV curves at different scan rates in nitrate and sulfate solutions of LIG/PI film electrodes. (**a**) Cd(NO_3_)_2_, (**b**) Co(NO_3_)_2_, (**c**) Ni(NO_3_)_2_, (**d**) CdSO_4_, (**e**) CoSO_4_ and (**f**) NiSO_4_.

Figure 6 illustrates the cyclic voltammetry (CV) curves of the LIG/PI thin film electrode under different scan rates in various nitrate and sulfate solutions. At scan rates of 5 to 20 mV s^−1^ in the Cd(NO_3_)_2_ solution, a quasi-rectangular shape was observed, illustrating stable capacitance behavior without a redox reaction. However, an oxidation peak appeared in CdSO_4_, indicating the possible decomposition of water. No obvious reduction peak from Cd^2+^ to Cd was detected due to the rapid scan. An obvious difference in Co(NO_3_)_2_ and CoSO_4_ was detected; however, a slight difference was observed in Ni(NO_3_)_2_ and NiSO_4_. Because the applied potential was higher (−1.2 V), both H^+^ and H_2_O participated in the reaction, reducing the pH of the solutions and increasing the conductivity.

**Figure 7:**
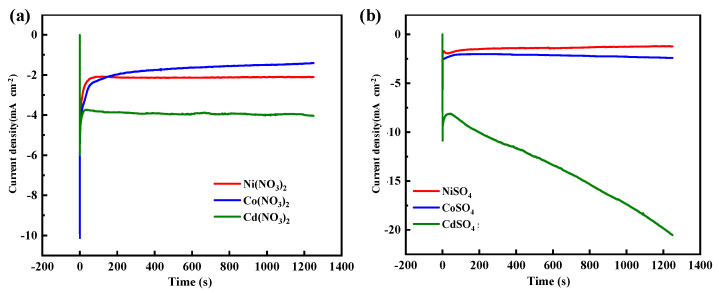
Current changes in (**a**) nitrate solution and (**b**) sulfate solution (−1.2 V).

After applying a potential of −1.2 V, the current density remained stable in the nitrate solution (Figure 7a). At the moment the voltage was applied, the current density surged in the nitrate solutions. Afterward, the current density decreased and stabilized. The stable current density values were approximately 1.5 mA cm^−^^2^ in the Co(NO_3_)_2_ solution, 2 mA cm^−^^2^ in the Ni(NO_3_)_2_ solution, and 4 mA cm^−^^2^ in the Cd(NO_3_)_2_ solution, respectively. Combining the changes in pH and conductivity, water decomposition occurred, and the generated H^+^ ions compensated for the adsorption of the Cd^2+^, Co^2+^, and Ni^2+^ ions, thereby ensuring that the mobility of the ions in the nitrate salt solutions remained unchanged. The current density was relatively stable in the NiSO_4_ (approximately 1.3 mA cm^−^^2^) and CoSO_4_ solution (approximately 2.3 mA cm^−^^2^). However, the current density in CdSO_4_ rapidly and linearly increased over time. This further illustrates that the stable reduction reaction in NiSO_4_ and CoSO_4_ was supported by the generated H^+^ ions. In addition, the reduction of Cd^2+^ ions was dramatically affected by the polarization.

When the voltage was −1.5 V, the current density exhibited changes as depicted in Figure S5. Except for CdSO_4_ and CoSO_4_, the current density changes in other solutions were consistent with the changes observed at −1.2 V, wherein the current density initially increased sharply before decreasing and ultimately stabilizing. The change was more pronounced in the CdSO_4_ solution, with a current density of 20 mA cm^−^^2^ at −1.2 V and 45 mA cm^−^^2^ at −1.5 V at 1200 s, respectively. In the CoSO_4_ solution, the current density did not remain stable but instead increased with time.

### 3.4. Adsorption Capacity

**Figure 8:**
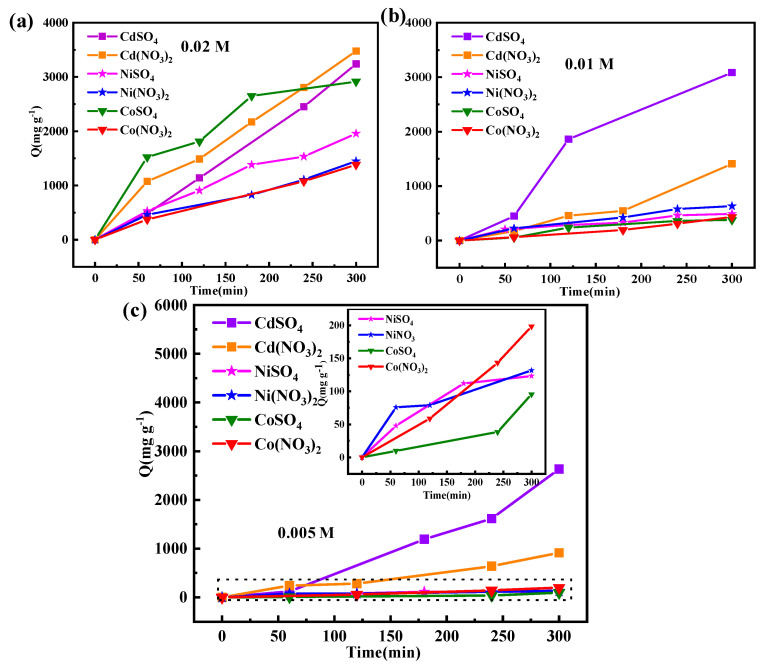
Adsorption of metal ions at different concentrations of nitrate and sulfate (−1.2 V). (**a**) 0.02 M, (**b**) 0.01 M, and (**c**) 0.005 M.

The adsorption amount of metal ions was measured using an atomic absorption spectrometer to evaluate the adsorption capability of the LIG/PI membrane electrode towards metal ions. We calculated Q and W using Formulas (1) and (2) (Supplementary Materials). The standard curves for different metal ions are depicted in Figure S6, while Figure 8 illustrates the temporal changes in the adsorption capacity. It was evident that with the increasing concentration, the adsorption capacity also increased. The adsorption capacity in Cd(NO_3_)_2_ increased from 913.5 mg g^−^^1^ at 0.005 M to 3479.8 mg g^−^^1^ at 0.02 M. Approximately 39% of Cd^2+^ ions were adsorbed on the electrode surface in 0.02M Cd(NO_3_)_2_ over 300 min. The adsorption capacity of 1381.5 mg g^−^^1^ in 0.02 M Co(NO_3_)_2_ solution was slightly lower than that of 1448.7 mg g^−^^1^ in Ni(NO_3_)_2_ solution. The adsorption capacity in the 0.005 M CdSO_4_ solution reached 2634.27 mg g^−^^1^, indicating the easy migration of Cd^2+^ and its reduction in the sulfate solution. Moreover, the adsorption capacity of 0.02 M CoSO_4_ and NiSO_4_ was also higher than that in the nitric acid solution. However, the recovery capability at a low concentration of CoSO_4_ and NiSO_4_ was similar. In sulfuric acid solution, the adsorption capacity followed the order of Cd ^2+^ > Co ^2+^ > Ni^2+^.

As the voltage was −1.5 V, the adsorption capacity also changed, as depicted in Figure S7. Approximately 40% Ni^2+^ in Ni(NO_3_)_2_ was covered in 100 min; then, the adsorption showed no obvious change due to the occupation of the active sites. It showed a faster adsorption with a longer adsorption time in Cd(NO_3_)_2_ than in Co(NO_3_)_2_. The recovery of Cd^2+^, Ni^2+^, and Co^2+^ was lower than approximately 20% when the concentration was 0.002 M. The recovery efficiency in the 0.002 M CoSO_4_ solution remained approximately 10%, while it increased in the NiSO_4_ and CdSO_4_ solution.

**Figure 9:**
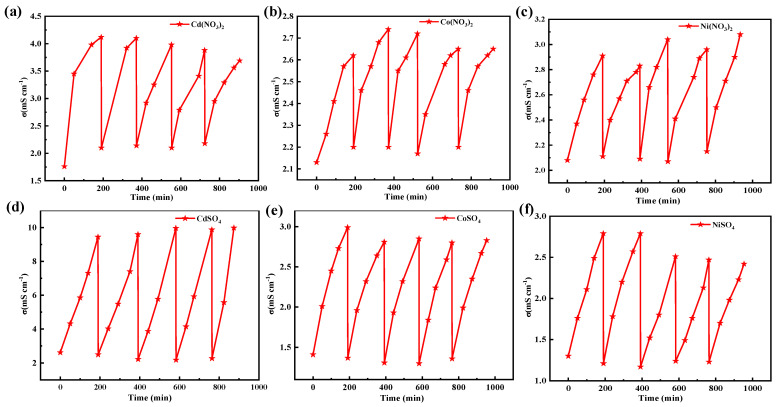
Cyclic performance of the electrodes. (**a**) Cd(NO_3_)_2_, (**b**) Co(NO_3_)_2_, (**c**) Ni(NO_3_)_2_, (**d**) CdSO_4_, (**e**) CoSO_4_, (**f**) NiSO_4_.

The recycling stability of the LIG electrode was one of the important factors for application. After the product was collected, which involved soaking in 5% nitric acid for 1 h, the electrode was washed with deionized water and then dried at 30 °C. The conductivities in this solution were stable in five adsorptions–desorption cycles, indicating that the LIG film possesses regenerative properties, as shown in Figure 9.

### 3.5. Reaction Mechanism

In nitrate, NO^3−^ ions migrate towards the counter electrode after the application of voltage, where they may acquire electrons and undergo reduction to form NO^2−^ ions (Equation (1)). Meanwhile, reaction Equation (2) occurs, generating H^+^ ions and reducing the pH of these solutions. Correspondingly, metallic ions swiftly migrate towards and combine with hydroxyl and carboxyl groups on the surface of the LIG film. Moreover, the generated OH^−^ ions further combine with metal ions to facilitate the formation of hydroxide precipitates (Equation (3)). However, water is decomposed in sulfates, and the acidic condition accelerates the reduction to form the corresponding metals (Equation (4)).

NO^3−^ + H_2_O + 2e^−^ → NO_2_^−^ + 2OH^−^ E = 0.93 V vs. SHE (1)

2H_2_O + 4e → O_2_ + 4H^+^ (2)

M^2+^ + 2OH^−^ → M(OH)_2_ (3)

M^2+^ + 2e → M (4)

### 3.6. Application of the Product

**Figure 10:**
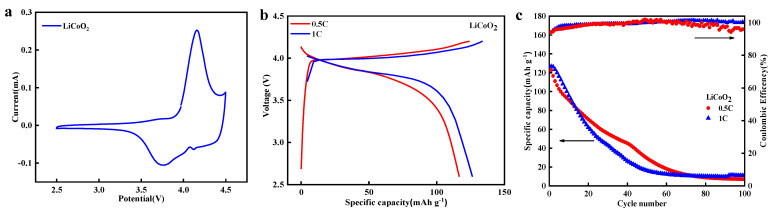
(**a**) CV curve, (**b**) charging/discharging curves, and (**c**) cyclic stability at 0.5 C and 1 C.

Taking recycled cobalt as an example, the investigation focused on the reutilization of the recovered product. The obtained Co(OH)_2_ served as the raw material for preparing LiCoO_2_. The CV curves (Figure 10) displayed a distinct redox peak at 4.1/3.7 V, corresponding to the insertion/extraction of Li. The specific capacity reached 122.8 mAh g^−^^1^ at a current density of 0.5 C, highly consistent with the reported data from commercially prepared materials [27]. The initial discharge capacity exhibited no significant change at a current density of 1 C. However, it rapidly decreased with increasing cycles, a phenomenon attributed to the particle size of the LiCoO_2_. In summary, the products recovered through this method also hold potential application value in other fields.

## 4. Conclusions

In this study, the transition metal ions, Co^2+^, Cd^2+^, and Ni^2+^, were recovered using LIG/PI thin films as the working electrodes through electrosorption and electrodeposition methods in nitrate and sulfate solutions, respectively. In nitrate and sulfate solutions, the LIG/PI electrode surface was found to accumulate hydroxides and dendritic metal deposits, respectively. After applying an external voltage, the pH of the solution decreased, while the conductivity increased. Compared to Co^2+^ and Ni^2+^, Cd^2+^ exhibited a lower standard electrode potential and stronger reducibility. Consequently, in both nitrate and sulfate solutions, the reaction sequence followed the order of Cd^2+^ > Co^2+^ > Ni^2+^, with the corresponding electrode adsorption quantities in the order of Cd^2+^ > Co^2+^ ~ Ni^2+^ in 0.005 to 0.02 M. Additionally, using the recovered Co(OH)_2_ as raw material, a LiCoO_2_ composite was prepared. The assembled battery with this composite exhibited a specific capacity of 122.8 mAh g^−1^, meeting practical application requirements. This has significance for fostering green development.

## Data Availability

The data presented in this study are available on request from the corresponding author.

## Supplementary Materials

The following supporting information can be downloaded at: https://www.mdpi.com/article/10.3390/ma17122965/s1, Figure S1. Changes in pH and conductivity during the electro adsorption process (−1.5 V). (a) Cd(NO_3_)_2_, (b) Ni(NO_3_)_2_, (c) Co(NO_3_)_2_, (d) CdSO_4_, (e) NiSO_4_, and (f) CoSO_4_. Figure S2. XRD pattern of electrode surface products in (a) nitric acid and (b) sulfate solutions with a voltage of −1.5 V. Figure S3. SEM of the product in (a,b) Co(NO_3_)_2_, (c,d) Ni(NO_3_)_2_, (e,f) Cd(NO_3_)_2_ solution (−1.5 V). Figure S4. SEM of the product in (a–c) CoSO_4_, (d–f) CdSO_4_, and (g–i) NiSO_4_ solution (−1.5 V). Figure S5. Current changes in nitrate solution and sulfate solution (−1.5 V). Figure S6. Standard Curve of (a) Cd(NO_3_)_2_, (b) Co(NO_3_)_2_, (c) Ni(NO_3_)_2_ solution (−1.2 V). Figure S7. Standard Curves of (a) Co(NO_3_)_2_, (b) Cd(NO_3_)_2_, (c) Ni(NO_3_)_2_ solution (−1.5 V), (d,e) Change in adsorption capacity over time at different concentrations, (f,g) Change in removal rate over time.
