# Supplementary material for: Recovery of Metal Ions (Cd2+, Co2+, and Ni2+) from Nitrate and Sulfate on Laser-Induced Graphene Film Using Applied Voltage and Its Application
Source: Materials (Basel). 2024 Jun 17;17(12):2965. doi: 10.3390/ma17122965 (PMC11205473; doi:10.3390/ma17122965)
Supplement: Supplementary file 1 [file materials-17-02965-s001.zip › materials-2994928-supplementary.pdf]

Supporting Information

Recovery of metal ions (Cd^{2+}, Co^{2+}, and Ni^{2+}) from nitrate and sulfate on laser-induced graphene film using applied voltage and its application

Xiu-man Wang, [1,2*] Tong Su, [3] Yujun Chai[3]

[1]State Key Laboratory of Mountain Bridge and Tunnel Engineering, Chongqing Jiaotong University, Chong qing, 400074, China

[2]School of Civil Engineering, Chongqing Jiaotong University, Chongqing, 400074, China

[3]College of Chemistry and Materials Science, Hebei Normal University, Hebei, Shijiazhuang 050024, China

*Correspondence: wangxiuman@cqjtu.edu.cn

Experimental

The adsorption capacity (Q) and removal rate (W) were calculated by Eq. 1 and Eq. 2, respectively.

$$Q = \frac{(C_0 - C_t)V}{m} \tag{1}$$

$$W = \frac{(C_0 - C_t)}{C_0} \times 100\% \tag{2}$$

where

Q is the amount of adsorption (mg g^{-1}) at the adsorption time t,

C_0 is the initial mass concentration of ions (mg L^{-1}),

C_t is the mass concentration of ions in the solution (mg L^{-1}) at the adsorption time t,

V is the volume of the solution (L),

m is the mass of the LIG film (g),

W is removal rate of ions (%) at the adsorption time t.

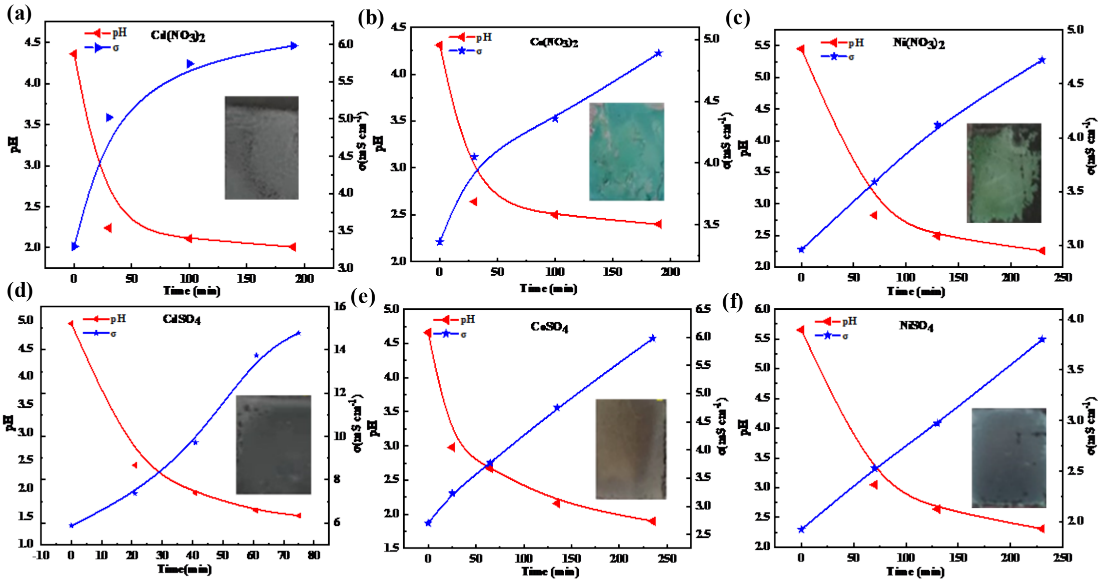

Figure S1. Changes in pH and conductivity during the electro adsorption process (-1.5 V). **(a)** $Cd(NO_3)_2$, **(b)** $Ni(NO_3)_2$, **(c)** $Co(NO_3)_2$, **(d)** $CdSO_4$, **(e)** $NiSO_4$, and **(f)** $CoSO_4$.

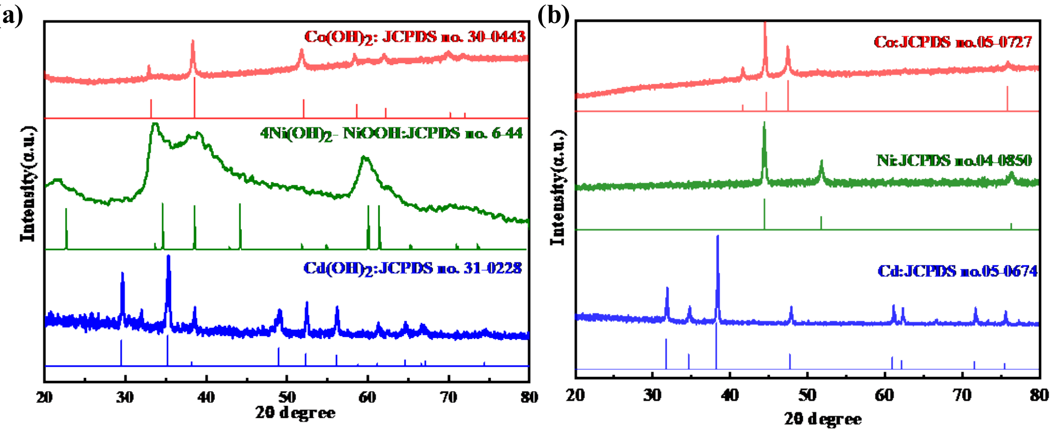

Figure S2. XRD pattern of electrode surface products in **(a)** nitric acid and **(b)** sulfate solutions with a voltage of -1.5 V.

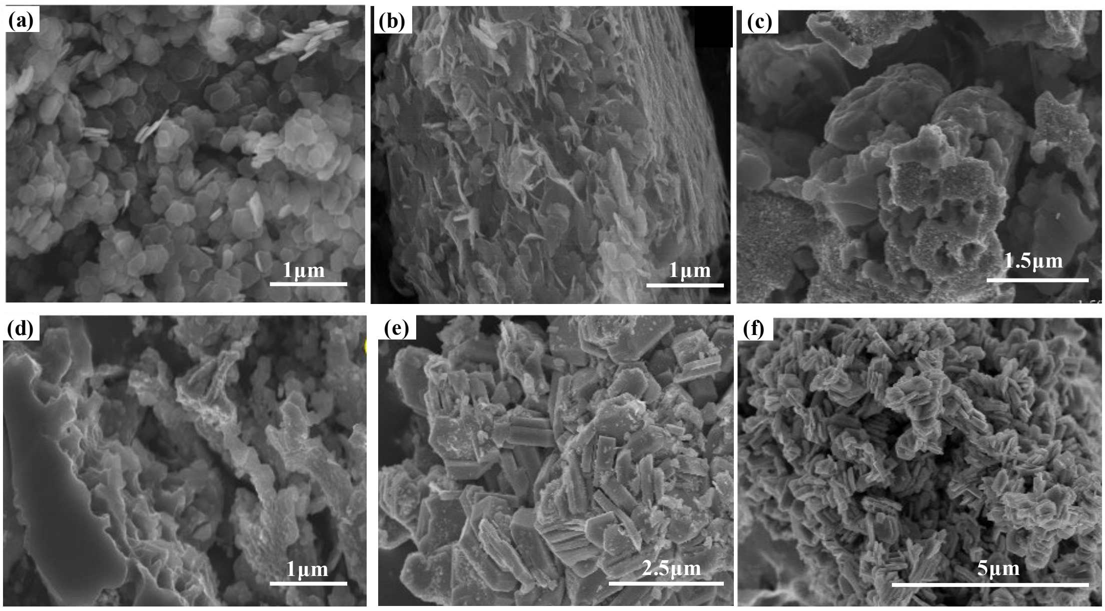

Figure S3. SEM of the product in **(a-b)** $Co(NO_3)_2$, **(c-d)** $Ni(NO_3)_2$, **(e-f)** $Cd(NO_3)_2$ solution (-1.5 V).

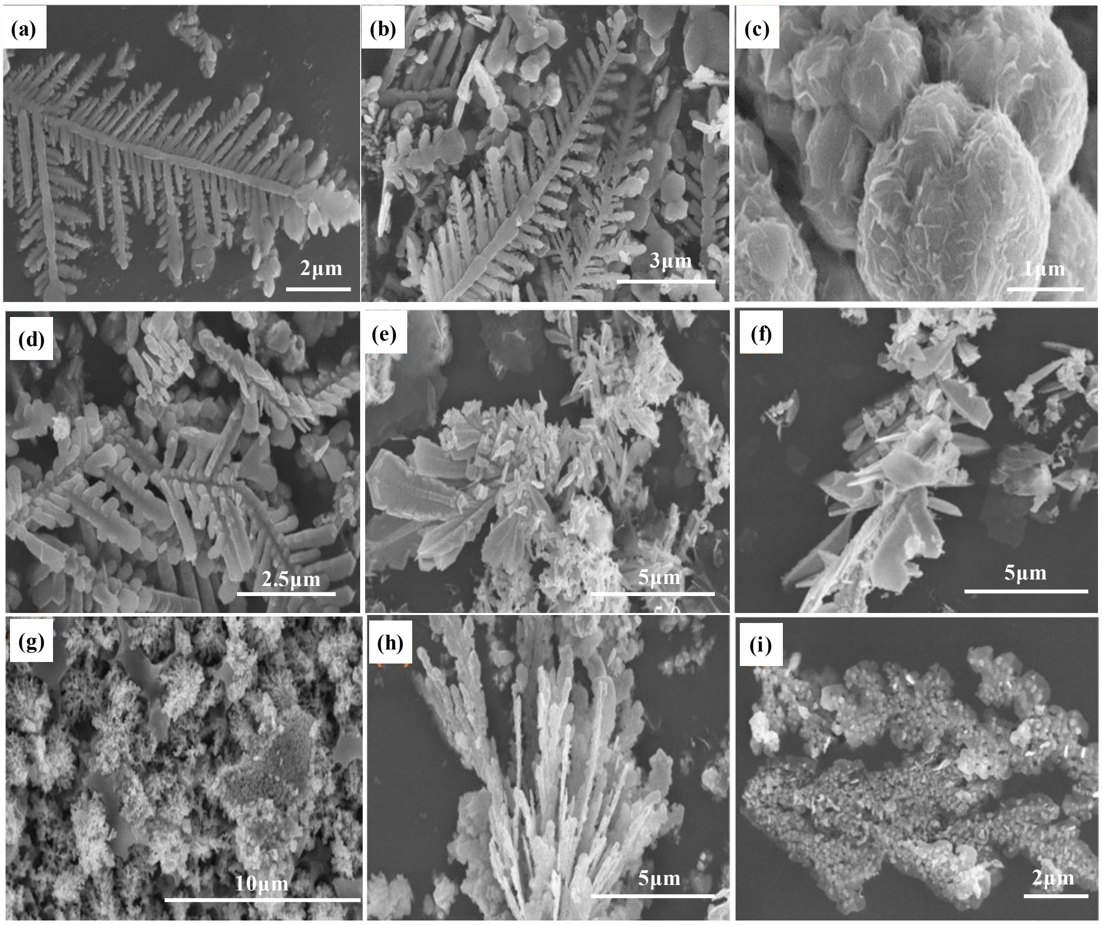

Figure S4. SEM of the product in **(a-c)** $CoSO_4$, **(d-f)** $CdSO_4$, and **(g-i)** $NiSO_4$ solution (-1.5 V).

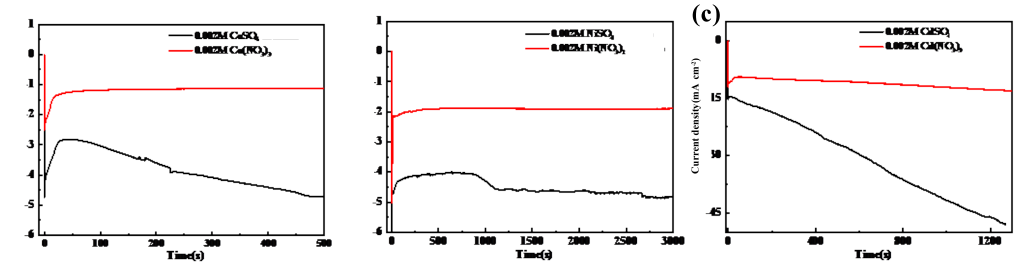

Figure S5. Current changes in nitrate solution and sulfate solution (-1.5 V).

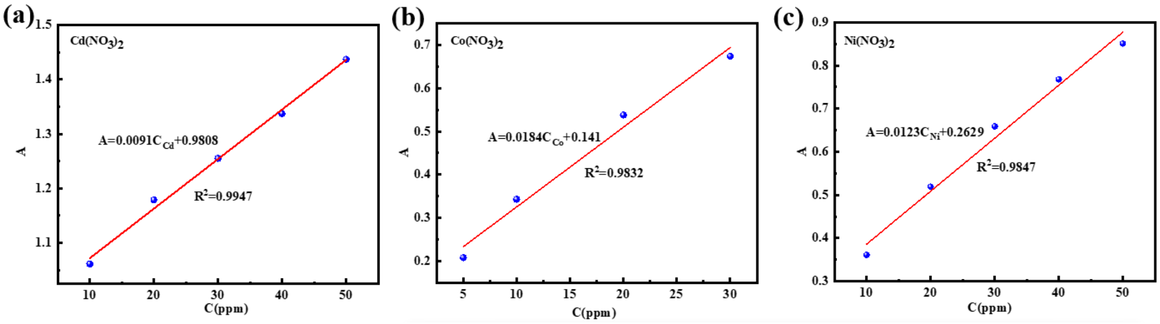

Figure S6. Standard Curve of **(a)** $Cd(NO_3)_2$, **(b)** $Co(NO_3)_2$, **(c)** $Ni(NO_3)_2$ solution (-1.2 V).

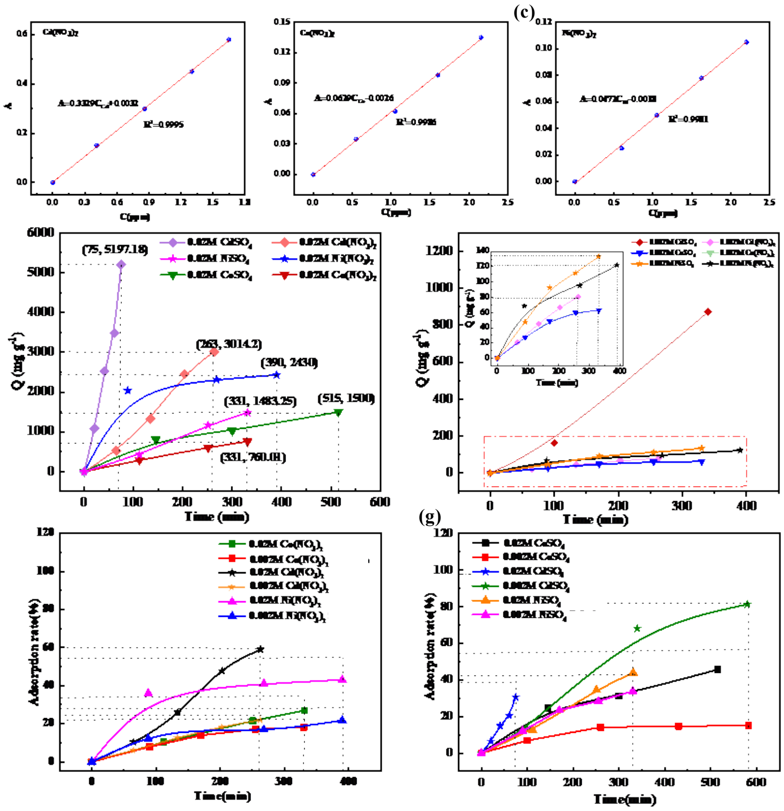

Figure S7. Standard Curves of **(a)** $Co(NO_3)_2$, **(b)** $Cd(NO_3)_2$, **(c)** $Ni(NO_3)_2$ solution (-1.5 V), **(d-e)** Change in adsorption capacity over time at different concentrations, **(f-g)** Change in removal rate over time.
